# The relationships of shift work, hair cortisol concentration and dyslipidaemia: a cohort study in China

**DOI:** 10.1186/s12889-022-14038-3

**Published:** 2022-08-29

**Authors:** Lejia Zhu, Yu Zhang, Lin Song, Ziqi Zhou, Jin Wang, Yangmei Wang, Lingli Sang, Jing Xiao, Yulong Lian

**Affiliations:** Department of Epidemiology and Medical Statistics, School of Public Health, Nantong University, Se Yuan Road, No. 9, Nantong, 226001 Jiangsu China

**Keywords:** Shift work, Dyslipidemia, Hair cortisol concentration, Mediating effect

## Abstract

**Background:**

Currently, cardiovascular disease is the leading cause of death, and dyslipidaemia is an independent and modifiable major risk factor. Previous studies on shift work with dyslipidaemia and hair cortisol concentration (HCC) have yielded conflicting results. The aim of this study was to clarify the association between shift work, dyslipidaemia, and HCC. We further explored the mediating effect of HCC.

**Methods:**

In this cohort study, baseline data were collected from participants in May 2013. The cohort included 2170 participants- 1348 shift workers and 822 non-shift workers- who were followed up for 6 years with four questionnaire surveys from July 2014, October 2015, and May to December 2019. Hair samples were collected from 340 participants during the baseline period for HCC testing with an automated radioimmunoassay. Dyslipidaemia was defined using the National Cholesterol Education Program Adult Treatment Panel III diagnostic criteria.

**Results:**

Shift workers had a higher risk of dyslipidaemia than workers on the fixed day shift (two-shift RR = 1.408, 95% CI: 1.102–1.798; three-shift RR = 1.478, 95% CI: 1.134–1.926; four-shift RR = 1.589, 95% CI: 1.253–2.015). Additionally, shift workers had higher HCC levels than fixed day shift workers, with geometric mean concentration (GMC) ± geometric standard difference (GSD) = 2.625 ± 2.012 ng/g, two-shift GMC ± GSD = 3.487 ± 1.930 ng/g, three-shift GMC ± GSD = 2.994 ± 1.813 ng/g, and four-shift GMC ± GSD = 3.143 ± 1.720 ng/g. High HCC was associated with a high incidence of dyslipidaemia. After controlling for confounding factors, this study showed that HCC played a role in mediating dyslipidaemia in shift workers and accounted for 16.24% of the effect.

**Conclusions:**

Shift work was linked to increased risk of dyslipidaemia compared with fixed day shift work. Higher HCC was associated with a higher prevalence of dyslipidaemia. HCC had a significant mediating effect on dyslipidaemia in shift workers.

## Introduction

The anciently high prevalence of dyslipidemia has been increasing in many developed and developing countries [1]. It exceeded 30% of adults in western countries [2]. An online survey of 65,892 people in Italy found that about 60% of participants had high blood cholesterol levels [3]. In the German population aged 18 to 79 years, the prevalence of dyslipidemia was 56.6% in men and 60.5% in women [4]. In Chinese adults, this was 40.4% [5].

The number of people doing shift work is probably between 10 and 25% of all employees [6]. Shift work has been reported to be associated with dyslipidemia, but published results are inconsistent [6–12]. Wu et al. [7] found that the number of people with dyslipidemia was much higher in shift work than that in non-shift work, and the difference was statistically significant. In a population-based study of 27,485 people, the youngest age group of shift workers was found to have low concentrations of high-density lipoprotein (HDL) cholesterol in both men and women [8], and a cross-sectional study found that shift work was a risk factor for lipid profile disturbances [6]. A prospective cohort study [9] reported that shift work with night shifts was associated with increased use of dyslipidemia medications after adjustments (HR = 1.33, 95% CI = 1.12–1.57). Dutheil, Frédéric et al. [11] found that shift work, and particularly permanent night shifts, was associated with dyslipidemia. However, EunKyo Kang [10] found that there were no significant differences in patients with dyslipidemia according to the type of shift. In a longitudinal study it was reported that the changes in total lipids (generic term for various lipid components in serum) caused by shift work were not statistically significant [12]. The influence of shift work on the incidence of dyslipidemia might be related to the irregular diet [13, 14] and high-sugar diet [15] caused by shift work. Moreover, sleep deprivation will increases the secretion of ghrelin, a growth-hormone-releasing acylated peptide from the stomach [16], which increases hunger and leads to obesity [17, 18] and dyslipidemia [19, 20].

Cortisol is a glucocorticoid hormone in the human hypothalamic-pituitary-adrenocortical (HPA) axis and is considered a retrospective biomarker for various chronic physiological and psychological stress diseases, anxiety, and depression [21]. Its secretion also fluctuates with the circadian rhythm [22]. HCC was more stable than the cortisol concentrations in blood, urine, and saliva [22]. The concentration of hair-like cortisol can be accumulated [23]; it can better reflect the long-term cortisol exposure level of the body [24]. Shift work may alter the circadian rhythm of the HPA axis and cause a long-term increase in cortisol concentration [25]. Chida and Steptoe [26] reported that the magnitude of the cortisol-awakening response was influenced by sleep deprivation. Manenschijn et al. [27] found that hair cortisol levels were significantly increased in individuals working in shifts, especially in the under 40 year old group (*P* < 0.01).

Current studies on the association between cortisol and dyslipidemia are inconsistent [28–31]. Cortisol concentration has been associated with dyslipidemia, and it has been suggested that chronic increased glucocorticoid was a secondary cause of dyslipidemia [28]. Pharmacological control of chronic glucocorticoid may have an effect on dyslipidemia [29]. Dhingra et al. [30] found that dyslipidemia was less common among Indian subjects with endogenous Cushing’s syndrome, which was caused by increased cortisol secretion. Correction of hypercortisolism may improve dyslipidemia in some patients for a few months. However a meta-analysis by Bancoset et al. [31] found that there was no significant improvement in dyslipidemia in patients with subclinical Cushing’s syndrome who underwent adrenalectomy. This reflects that cortisol has no association with dyslipidemia.

This study aimed to elucidate the relationship between shift work, HCC, and dyslipidemia, and to further explore the mediating effect of HCC. We investigated different shift patterns separately, with the hypothesis that: (1) shift work may cause dyslipidemia, and different shift patterns may have different effects on the incidence of dyslipidemia; (2) higher HCC level is associated with increased incidence of dyslipidemia; (3) HCC was a mediator between shift work and dyslipidemia.

## Methods

### Study population

Participants were selected between May and December 2013 using a multistage cluster and stratified random sampling. All cities in Xinjiang with administrative bureaus and petrochemical companies listed in the China Petroleum and Petrochemical Species Classification Catalog were identified, and one city was selected randomly. Five petrochemical companies in one city were randomly selected, and all the employees in each company were divided into 4 groups, giving a total of 20 groups. All groups were numbered, and 10 groups were randomly selected according to the random number table method. A total of 3400 were selected for study. They underwent health examination in 2013 at Karamay City Center for Disease Control and Prevention in Xinjiang and filled out a questionnaire with basic information. Those eligible for the study were employees of the Karamay City Petroleum Administration and Petrochemical Company who had been employed in that position for 1 year, were 20–60 years old, and had signed an informed consent form. Patients with dyslipidaemia at baseline (*n* = 1037), diseases affecting blood lipids, medications affecting blood lipids, or diet (*n* = 52), or hair shorter than 3 cm (*n* = 11) were excluded. Those who answered less than 80% of the questions in the questionnaire (*n* = 48), left work, and were unavailable during follow-up (*n* = 69) were also excluded. The survey was launched in May 2013 and included a 6-year follow-up period during which participants did not change their shift work. Participants were followed up with questionnaires and occupational health examinations at the Karamay Center for Disease Control and Prevention in Xinjiang from May to December 2014, 2015, and 2019. The study cohort included 2170 participants, 1021 men and 1149 women; 1348 were shift workers and 822 were non-shift workers.

In the early stage of the study, we regarded the shift population as the exposed group, with an incidence rate of p1 = 0.238, and the general adult population as the non-exposed group, with an incidence rate of p0 = 0.186 [32, 33]. Take the test level α = 0.05, the power of the test was 1- β (take β = 0.10). The formula for calculating the sample size was as follows:

$$n=\frac{{\left({z}_{1-\alpha /2}\sqrt{2\overline{pq}}+{Z}_{\beta}\sqrt{p_0{q}_0+{p}_1{q}_1}\right)}^2}{{\left({p}_1-{p}_0\right)}^2}$$

The required sample size was calculated to be 1297. The sample size in this study met these requirements.

### Shift work

We used a self-reported questionnaire to obtain information on shift work patterns, family medical history, and personal information such as smoking and drinking. Employees who regularly worked fixed-day shifts from 8:00 am to 5:00 pm were considered non-shift workers. Employees who worked night shifts were considered shift workers and were divided into two, three, and four shifts as described below. “Two shifts” included two 12-hour shifts and two groups of workers alternating weekly; “Three shifts” included two 12-hour shifts with three groups of workers alternating weekly, with one of the groups resting; “Four shifts” included three 8-hour shifts (morning, mid, and evening) with four groups of workers working alternately and with one at group at rest. Shift work was thus divided into four groups: fixed day shift, two shifts, three shifts, and four shifts.”

### Dyslipidemia

Blood lipid data was obtained at annual occupational health examinations. Dyslipidemia was determined by measuring the concentration of cholesterol in the four lipoproteins, total cholesterol (TC), triglycerides (TG), low-density lipoprotein cholesterol (LDL-C), and high-density lipoprotein cholesterol (HDL-C) [34]. In participants, dyslipidemia required one of the following results in two assays performed 2 weeks apart, TC > 5.18 mmol/L, TG > 1.7 mmol/L, and HDL-C < 1.04 mmol/L within 2 weeks meet conditions [5].

### HCC

During the baseline period, we randomly divided 2170 participants into 70 groups of 31 subjects each, and randomly selected 11 of these groups, to collect hair samples from a total of 341 subjects to collect hair samples. Researchers reported that natural hair color had no effect on hair cortisol concentrations [35], and Sauvé et al. found that chemically treated hair (dyed hair) to have significantly lower hair cortisol concentrations than untreated hair [36]. Finally, after deleting 5 maximum and 4 minimum values and 1 unnatural hair color, the hair cortisol concentrations of 331 subjects were included in the analysis.

Hair samples (2–3 cm, 20–30 mg) were collected from the hair roots of the participants. Pretreatment of hair samples was performed according to the experimental protocol described in the patent, “Pretreatment method for detecting cortisol content in hair” [37]. The hair sample was soaked with 2–3 ml of isopropyl alcohol for 5 minutes, washed and peeled, then frozen in liquid nitrogen for more than 4 hours and then pulverised. The pulverised hair sample was placed in a centrifuge tube, mixed with 5 ml of methanol solution and 3 ml of ether solution, and placed in a water bath at 50.8 °C for 16 h for extraction and incubation. During analysis, the hair fragments were mixed by multiple inverting and centrifuged at low speed at 3500 rpm for 15 min. The supernatant was transferred to a 4 ml Eppendorf tube, and the extracted mixture was dried with a nitrogen blower. After the addition of 2 ml of phosphate buffer solution, the sample was stored at − 4 °C in a refrigerator until the day of testing. HCC was detected using an automated radioimmunoassay.

### Covariates

Covariates included sex, age, body mass index (BMI, kg/m^2^), ethnicity, marital status, education level, family history of hypertension, coronary heart disease, stroke, diabetes, income level (Yuan), job tenure (years), type of work, smoking, drinking, and exercise. Participants were stratified by age (youth group: 20–29 years, young and middle-aged group: 30–39 years, and middle-aged and elderly group: 40–60 years), BMI (Chinese standard BMI value: low body weight: < 18.5 kg/m^2^, normal weight: 18.5–23.9 kg/m^2^, overweight: 24–28.0 kg/m^2^, and obese ≥28 kg/m^2^ [38]). Ethnicity was divided into “Han”, “Uygur” and “other minority”. Marital status was divided into ‘not married’, ‘married’, and ‘other’ (divorced, widowed, or remarried, respectively). The educational level was divided into ‘high school or below’, ‘junior college education’, and ‘college or above’. A family history of hypertension was subdivided into ‘yes’, ‘no’, or ‘unknown’. A family history of coronary heart disease was subdivided into ‘yes’, ‘no’, or ‘unknown’. Family history of stroke was classified as ‘yes’, ‘no’, or ‘unknown’. Family history of diabetes was divided into ‘yes’, ‘no’, or ‘unknown’. The income level (Yuan) was divided into ‘< 3000/$422’, ‘3000-5000/$422–$736’, and ‘>5000/$736’ Yuan. Job tenure was divided into ‘< 10’, ‘10–20’, and ‘≥ 20’ years. The type of work was divided into ‘oil’, ‘oil recovery’, ‘refining’, and ‘other’. Smoking was divided into ‘often’ (≥1 cigarette/day), ‘occasional’ (< 1 cigarette/day), ‘quit smoking’, and ‘nonsmoking’. Drinking was divided into ‘often’ (≥ 8 g/day), ‘occasional’ (< 8 g/day), ‘quit drinking’, and ‘nondrinking’. Physical exercise was divided into ‘no exercise’, ‘< 3 times/week’, ‘≥ 3 times/week’, and ‘irregular’.

### Statistical analysis

EpiData3.0, the questionnaire’s double-track data entry software, and STATA13.0 were used to organise and analyse the data. Measurement data were described as mean average (¯X) ± standard deviation (SD) or median and interquartile range [M(Q1-Q3)] and geometric mean concentrations (GM) ± the GSD to improve statistical power. Comparison of measured data was performed using the t-test or analysis of variance, and comparison of count data was performed by χ^2^ test. Four models were established to perform logistic regression analysis between indicators. Model 1 represented associations between indicators without adjustment for confounders, and Model 2 was adjusted for gender, age, ethnicity, marital status, education level, type of work, length of service, and average monthly income. Model 3 was adjusted for smoking status, drinking status, physical exercise, and BMI based on Model 2. Model 4 was adjusted on the basis of Model 3 for hypertension, coronary heart disease, stroke, and family history of diabetes. Linear regression was used to analyse the association between HCCs and changes in blood lipid levels. HCC values showed a skewed distribution. Make HCC values normally distributed by log transformation. Shift work was divided into five groups according to shift pattern and a fixed day shift as a reference group.

We conducted a mediating-effect analysis to understand the mechanism by which one variable affects another. The coefficient between shift work and dyslipidemia was the overall effect. When HCC was the mediator, the coefficient between shift work and dyslipidemia represented a direct influence. The mediation effect was calculated by subtracting the direct effect from the total effect [39]. Previous studies have shown that excessive HCC may have an effect on dyslipidemia [40]. Methods described by Karlson, Holm, and Brin [41] were used to verify the significance of the HCC effect. If both the overall effect and the indirect effect were significant and the direct effect was not, then HCC was considered to regulate the relationship between shift work and dyslipidemia [42]. However, if all the effects were significant, then HCC was considered to have played a role in mediating the outcome [43]. We used this method to estimate the percentage of the total effect mediated by HCC.

### Ethical considerations

All participants signed an informed consent form after receiving information about the study. This study was approved by the Nantong University Ethics Committee (2013-L073).

## Results

A total of 2170 subjects were included in this research cohort, whose ages ranged from 20 to 60 years (37.86 ± 7.56 years), including 1021 men (47.05%) and 1149 women (52.95%). There were 1348 employees who worked in shifts, representing 62.1% of the total population. The proportion of shift workers aged 20–29 and 30–40 was significantly higher than that of non-shift workers (20–29: 12.65%, 30–39: 32.48%), at 18.25 and 36.05%, respectively, while the proportion of those aged 40–60 was significantly lower than that of non-shift workers. Of the shift workers, 24.33% had a working age of less than 10 years, which was significantly higher than the 19.83% of regular day shift workers, while 49.70% of shift workers had been working for ≥20 years, which was significantly lower than the 55.72% of regular day shift workers. These differences were statistically significant. There were statistically significant differences in the distribution of shift workers and non-shift workers across different types of work and different drinking frequencies. There were no significant differences in the distribution of shift workers by sex, age, ethnicity, marital status, education level, etc. (Table 1).

**Table 1:** Different demographic characteristics of shift work and dyslipidemia in Karamay, Xinjiang in 2013

	Variable	Shift work ***N*** = 1348(62.1%) n%	Non-Shift work ***N*** = 822 (37.9%) N%	***P***	Dyslipidaemia ***N*** = 696 (32.07%) n%	Non-Dyslipidaemia ***N*** = 1472 (67.93%) n%	***P***
**Sex**	**Male**	626 (46.44)	395 (38.7)	0.465	407 (58.48)	614 (41.71)	< 0.001
	**Female**	722 (53.56)	427 (37.2)		289 (41.52)	860 (58.42)	
**Age**	**20–29**	246 (18.25)	104 (12.65)	0.006	88 (12.64)	262 (17.80)	0.009
	**30–39**	486 (36.05)	267 (32.48)		247 (35.49)	506 (34.38)	
	**40–60**	616 (45.70)	451 (54.86)		361 (51.87)	706 (47.96)	
**BMI (kg/m2)**	**<18.5**	44 (3.26)	25 (3.04)	0.661	10 (1.44)	59 (4.01)	< 0.001
	**18.5–23.9**	710 (52.67)	429 (52.19)		284 (40.80)	855 (58.08)	
	**24–28.0**	453 (33.61)	268 (32.60)		267 (38.36)	454 (30.84)	
	**≥28**	141 (10.46)	100 (12.17)		135 (19.40)	106 (7.20)	
**Ethnicity**	**Han**	1100 (81.60)	678 (82.48)	0.624	551 (79.12)	1227 (83.36)	0.67
	**Uygur**	180 (13.35)	110 (13.38)		106 (15.23)	184 (12.5)	
	**Other minority**	68 (5.05)	34 (4.14)		39 (5.60)	63 (4.28)	
**Marital status**	**Not married**	193 (14.32)	95 (11.56)	0.175	88 (12.64)	200 (13.59)	0.618
	**Married**	988 (73.29)	618 (75.18)		506 (72.70)	1100 (74.73)	
	**Other (divorced, widowed, remarried)**	167 (12.39)	109 (13.26)		102 (14.66)	174 (11.82)	
**Education level**	**High school or below**	363 (26.93)	241 (29.32)	0.180	200 (28.74)	404 (27.45)	0.577
	**Junior college education**	760 (56.38)	466 (56.69)		382 (54.89)	844 (57.34)	
	**College or above**	225 (16.70)	115 (13.99)		114 (16.38)	226 (15.35)	
**Family history of hypertension**	**Yes**	578 (42.88)	360 (43.80)	0.722	344 (49.43)	594 (40.35)	< 0.001
	**No**	685 (50.82)	405 (49.27)		297 (42.67)	793 (53.87)	
	**Unknown**	85 (6.30)	57 (6.93)		55 (7.90)	87 (5.91)	
**Family history of coronary heart disease**	**Yes**	301 (22.33)	156 (18.98)	0.178	257 (36.93)	200 (13.59)	< 0.001
	**No**	936 (69.44)	595 (72.38)		387 (55.60)	1144 (77.72)	
	**Unknown**	111 (8.23)	71 (8.64)		52 (7.47)	130 (8.83)	
**Family history of stroke**	**Yes**	117 (8.68)	65 (7.91)	0.289	121 (17.39)	61 (4.14)	< 0.001
	**No**	1092 (81.01)	687 (83.58)		480 (68.97)	1299 (88.25)	
	**Unknown**	139 (10.31)	70 (8.52)		95 (13.65)	114 (7.74)	
**Family history of diabetes**	**Yes**	364 (27.00)	202 (24.57)	0.241	263 (37.79)	303 (20.58)	< 0.001
	**No**	866 (64.24)	557 (67.76)		353 (50.72)	1070 (72.69)	
	**Unknown**	118 (8.75)	63 (7.66)		80 (11.49)	101 (6.86)	
**Income level (yuan)**	**< 3000/$422**	269 (19.96)	165 (20.07)	0.712	142 (20.40)	292 (19.84)	0.002
	**3000–5000/$422–$736**	938 (69.58)	580 (70.56)		462 (66.38)	1056 (71.74)	
	**> 5000/>$736**	141 (10.46)	77 (9.37)		92 (13.22)	126 (8.56)	
**Job tenure**	**< 10**	328 (24.33)	163 (19.83)	0.014	152 (21.84)	339 (23.03)	0.590
	**10–20**	350 (25.97)	201 (24.45)		186 (26.72)	365 (24.80)	
	**≥20**	670 (49.70)	458 (55.72)		358 (51.4)	770 (52.31)	
**Type of work**	**Oil**	391 (29.01)	260 (31.63)	0.002	297 (42.67)	354 (24.05)	< 0.001
	**Oil recovery**	212 (15.73)	128 (15.57)		99 (14.22)	241 (16.37)	
	**Refining**	143 (10.61)	123 (14.96)		51 (7.33)	215 (14.61)	
	**Other**	602 (44.66)	311 (37.83)		249 (35.78)	664 (45.11)	
**Smoking**	**Often**	274 (20.33)	183 (22.26)	0.683	159 (22.85)	298 (20.24)	0.087
	**Occasional**	216 (16.02)	123 (14.96)		115 (16.52)	224 (15.22)	
	**Quite smoking**	70 (5.19)	39 (4.75)		42 (6.03)	67 (4.55)	
	**Nonsomking**	788 (58.46)	477 (58.03)		380 (54.60)	885 (60.12)	
**Drinking**	**Often**	210 (15.58)	161 (19.59)	0.021	135 (19.40)	236 (16.03)	0.001
	**Occasional**	470 (34.87)	249 (30.29)		248 (35.63)	471 (32.00)	
	**Quite drinking**	82 (6.08)	40 (4.87)		47 (6.75)	75 (5.10)	
	**Nondrinking**	586 (43.47)	372 (45.25)		266 (38.22)	692 (47.01)	
**Physical exercise**	**No exercise**	354 (26.61)	219 (26.64)	0.756	215 (30.89)	358 (24.32)	0.010
	**< 3 Times/week**	443 (32.86)	264 (32.12)		219 (31.47)	488 (33.15)	
	**≥3 Times/week**	204 (15.13)	114 (13.87)		90 (12.93)	228 (15.49)	
	**Irregular**	347 (25.74)	225 (27.37)		172 (24.71)	400 (27.17)	

A total of 696 patients developed dyslipidaemia during the study period. The incidence of dyslipidaemia was 32.07% (95% CI: 30.11–34.03). The incidence was 39.86% in men and 25.15% in women (*P* < 0.001). Different age groups; BMI; monthly income; type of work; alcohol consumption; physical exercise; and family history of hypertension, coronary heart disease, stroke, and diabetes had statistically significant (*p* < 0.05) differences in the prevalence of dyslipidemia. No significant difference in the prevalence of dyslipidemia among ethnicity, marital status, education level, working age, and smoking was observed (Table 1).

Analysis of the relationship between different shift patterns and dyslipidaemia showed that the incidence of dyslipidaemia in the second shift (RR = 1.408, 95% CI: 1.102–1.798), third shift (RR = 1.478, 95% CI: 1.134–1.926), and fourth shift (RR = 1.589, 95% CI = 1.253–2.015) were significantly higher than those in the fixed day shifts (*P* < 0.05). After adjustment for all confounding factors, the risk of dyslipidaemia was still significantly higher in workers on two-shift (RR = 1.341, 95% CI: 1.10–1.781), three-shift (RR = 1.560, 95% CI: 1.152–2.111), and four-shift (RR = 1.782, 95% CI: 1.359–2.336) than in workers on fixed day shifts. No significant differences were found between the HCC in the three-shift group versus the fixed day-shift group and in the four-shift group versus the two-shift group (Table 2).

**Table 2:** Logistic regression analysis of the influence of different shift patterns on the incidence of dyslipidaemia in Karamay, Xinjiang from 2013 to 2019

Shift Work	Dyslipidaemia n (%)	Model 1		Model 2		Model 3		Model 4	
		RR (95%CI)	***P***	RR (95%CI)	***P***	RR (95%CI)	***P***	RR (95%CI)	***P***
Fixed day shift	220 (26.8)	1.000		1.000		1.000		1.000	
Two shifts	161 (34.0)	1.408 (1.102–1.798)	0.006	1.465 (1.131–1.897)	0.004	1.461 (1.119–1.908)	0.005	1.341 (1.010–1.781)	0.043
Three shifts	128 (35.1)	1.478 (1.134–1.926)	0.004	1.550 (1.175–2.044)	0.002	1.654 (1.244–2.200)	0.001	1.560 (1.152–2.111)	0.004
Four shifts	187 (36.7)	1.589 (1.253–2.015)	<0.001	1.756 (1.369–2.254)	< 0.001	1.820 (1.408–2.352)	< 0.001	1.782 (1.359–2.336)	< 0.001

Model 1: Represent the association between shift work and dyslipidaemia without the adjustment of confounding factors

Model 2: Adjusted for sex, age, ethnicity, marital status, education level, type of work, length of service, and average monthly income

Model 3: Adjusted for smoking, drinking, physical exercise, and BMI based on Model 2

Model 4: Adjusted for family history of hypertension, coronary heart disease, stroke**,** and diabetes based on Model 3

*RR：* relative risk

During the baseline period, there were no significant differences in blood lipid levels between workers on the different shifts. Blood lipid levels at baseline and at the end of follow-up were compared between workers in the different shifts. It was found that the TC, TG, and LDL-C levels of the workers were significantly increased, while HDL-C levels were significantly decreased in all shifts. At the end of follow-up, the TC levels of workers in two-shift (5.042 ± 1.009 mmol/L), three-shift (5.052 ± 0.961 mmol/L), and four-shift (5.268 ± 0.942 mmol/L) were significantly higher than those of workers in regular day shifts (4.810 ± 0.738 mmol/L) (*p* < 0.01). Three-shift (2.864 ± 0.753 mmol/L), and four-shift (2.914 ± 0.768 mmol/L) had significantly higher LDL-C levels than workers in the regular day-shift (2.730 ± 0.615 mmol/L) (*p* < 0.01). There were no significant differences in the levels of TG and HDL-C among workers in each shift mode (Table 3).

**Table 3:** Effects of different shift patterns on blood lipid levels in Karamay, Xinjiang from 2013 to 2019

	Shiftwork	Baseline	End	D-value	***P***
**TC (**$\overline{\boldsymbol{X}}$ **±SD, mmol/L)**	**Fixed day shift**	4.539 ± 0.703	4.810 ± 0.738a	0.270 ± 0.711a	< 0.001
	**Two shifts**	4.515 ± 0.699	5.042 ± 1.009b	0.527 ± 1.025bc	< 0.001
	**Three shifts**	4.571 ± 0.708	5.052 ± 0.961b	0.481 ± 0.910b	< 0.001
	**Four shifts**	4.608 ± 0.729	5.268 ± 0.942c	0.660 ± 0.994c	< 0.001
	**P**	0.172	< 0.001	< 0.001	–
**TG (**$\overline{\boldsymbol{X}}$ **±SD, mmol/L)**	**Fixed day shift**	1.142 ± 0.458	1.348 ± 0.790	0.206 ± 0.779	< 0.001
	**Two shifts**	1.150 ± 0.459	1.410 ± 1.892	0.260 ± 1.898	0.003
	**Three shifts**	1.158 ± 0.494	1.352 ± 0.909	0.237 ± 0.777	< 0.001
	**Four shifts**	1.125 ± 0.478	1.409 ± 1.160	0.284 ± 1.151	< 0.001
	**P**	0.690	0.725	0.069	–
**HDL-C (**$\overline{\boldsymbol{X}}$ **±SD, mmol/L)**	**Fixed day shift**	1.373 ± 0.248	1.301 ± 0.281	−0.072 ± 0.287	< 0.001
	**Two shifts**	1.358 ± 0.241	1.271 ± 0.260	−0.086 ± 0.272	< 0.001
	**Three shifts**	1.389 ± 0.269	1.311 ± 0.305	−0.078 ± 0.295	< 0.001
	**Four shifts**	1.394 ± 0.261	1.312 ± 0.301	−0.082 ± 0.318	< 0.001
	**P**	0.110	0.104	0.846	–
**LDL-C (**$\overline{\boldsymbol{X}}$ **±SD, mmol/L)**	**Fixed day shift**	2.660 ± 0.621	2.730 ± 0.615a	0.069 ± 0.617a	0.001
	**Two shifts**	2.613 ± 0.592	2.796 ± 0.738ab	0.184 ± 0.745b	< 0.001
	**Three shifts**	2.687 ± 0.632	2.864 ± 0.753b	0.177 ± 0.728b	< 0.001
	**Four shifts**	2.691 ± 0.634	2.914 ± 0.768b	0.223 ± 0.794b	< 0.001
	**P**	0.197	< 0.001	0.001	–

^a,b,c^:There was no statistically significant difference in blood lipid levels between groups marked with the same letter

D-value: Mean of differences, mean of baseline and end differences

Finally, in the baseline period, the HCCs of 331 subjects were included in the analysis. HCC of males (3.651 ± 2.071 ng/g) was significantly higher than that of females (2.588 ± 1.712 ng/g); HCC of oil transport workers (3.276 ± 1.881 ng/g) was significantly higher than that of oil recovery workers (2.503 ± 1725 ng/g), refinery workers (2721 ± 2029 ng/g) and others (2683 ± 1883 ng/g); HCC of non-smoking workers (2777 ± 1.847 ng/g) was significantly lower than that of regular smokers (3.156 ± 1.914 ng/g), occasional smokers (3.279 ± 1.936 ng/g), and smokers who had quit (4.276 ± 2.177 ng/g); these differences were all statistically significant. Hair cortisol concentrations were not statistically different for other demographic characteristics (Table 4).

**Table 4:** Comparison of general demographic characteristics and hair cortisol concentration levels in Karamay, Xinjiang in 2013

Variable	n	HCC M(Q_1_-Q_3_) (ng/g)	HCC GM ± GSD (ng/g)	t/F	*p*
Sex					
male	132	3.744 (2.363–6.298)	3.651 ± 2.071	4.653	< 0.001
female	199	2.584 (1.876–3.786)	2.588 ± 1.712		
Age					
20–29	52	3.037 (1.962–4.659)	3.058 ± 1.842	0.173	0.918
30–39	131	2.792 (2.116–4.821)	3.009 ± 1.916		
40–60	148	2.868 (1.884–4.358)	2.903 ± 1.912		
BMI (kg/m^2^)					
<18.5	11	2.687 (2.134–3.611)	2.773 ± 1.575	0.948	0.426
18.5–23.9	174	2.804 (1.876–4.447)	2.865 ± 1.840		
24–28.0	103	2.744 (1.947–4.857)	3.038 ± 2.134		
≥ 28	43	3.279 (2.357–4.021)	3.302 ± 1.638		
Ethnicity					
Han	258	2.830 (1.906–4.540)	2.909 ± 1.930	1.433	0.24
Uygur	43	2.933 (2.128–4.289)	2.942 ± 1.762		
Other minority	30	3.561 (2.361–5.570)	3.585 ± 1.807		
Marital status					
Not married	41	3.114 (1.899–4.878)	2.962 ± 1.758	1.014	0.364
Married	249	2.853 (2.081–4.440)	3.035 ± 1.899		
Other (divorced, widowed, remarried)	41	2.735 (1.551–4.374)	2.602 ± 2.036		
Education level					
High school or below	75	2.722 (1.860–3.611)	2.666 ± 1.760	1.672	0.189
Junior college education	210	3.022 (2.075–4.909)	3.109 ± 1.961		
College or above	46	2.887 (1.741–4.168)	2.8671.839		
Family history of hypertension					
Yes	114	2.895 (1.942–4.759)	2.97 ± 1.964	0.049	0.953
No	195	2.878 (1.957–4.1611)	2.980 ± 1.871		
Unknown	22	2.761 (1.876–4.633)	2.849 ± 1.875		
Family history of coronary heart disease					
Yes	49	2.773 (1.886–5.178)	3.152 ± 1.878	0.359	0.699
No	262	2.890 (1.966–4.275)	2.953 ± 1.906		
Unknown	20	2.354 (1.837–4.788)	2.755 ± 1.917		
Family history of stroke					
Yes	49	2.669 (2.029–3.206)	2.692 ± 1.514	0.323	0.724
No	262	2.906 (1.969–4.494)	2.999 ± 1.916		
Unknown	20	2.354 (1.850–4.902)	2.771 ± 1.897		
Family history of diabetes					
Yes	59	2.773 (1.969–4.763)	3.060 ± 1.750	0.083	0.92
No	248	2.889 (1.924–4.359)	2.946 ± 1.945		
Unknown	24	3.184 (1.945–4.788)	2.868 ± 1.952		
Income level (yuan)					
< 3000/<$422	74	2.850 (1.932–4.328)	2.827 ± 1.952	0.536	0.57
3000–5000/$422–$736	233	2.853 (1.938–4.440)	2.978 ± 1.847		
> 5000/>$736	24	3.116 (2.129–7.319)	3.310 ± 2.271		
Job tenure (years)					
< 10	93	3.105 (2.213–4.794)	3.012 ± 1.770	0.032	0.968
10–20	94	2.736 (2.004–4.578)	2.946 ± 1.913		
≥ 20	144	2.965 (1.918–4.282)	2.956 ± 1.911		
Type of work					
Oil	176	3.043 (2.116–5.135)	3.276 ± 1.881^a^	3.129	0.026
Oil recovery	34	2.616 (1.625–3.639)	2.503 ± 1.725^b^		
Refining	49	2.816 (1.795–4.506)	2.721 ± 2.029^b^		
Other	72	2.821 (1.768–4.078)	2.683 ± 1.883^b^		
Smoking					
Often	63	3.471 (2.158–4.936)	3.156 ± 1.914^c^	3.163	0.025
Occasional	40	3.438 (2.148–5.228)	3.279 ± 1.936^c^		
Quit smoking	17	3.786 (2.390–8.302)	4.276 ± 2.177^c^		
Nonsmoking	211	2.692 (1.910–3.970)	2.777 ± 1.847^d^		
Drinking					
Often	22	4.025 (2.252–7.745)	3.832 ± 2.250	1.71	0.178
Occasional	130	3.152 (1.948–4.822)	3.095 ± 1.912		
Quit drinking	16	2.688 (2.141–6.508)	3.178 ± 2.147		
Nondrinking	163	2.722 (1.917–4.000)	2.755 ± 1.805		
Physical exercise					
No exercise	66	2.704 (1.812–3.779)	2.713 ± 1.779	2.262	0.081
<3 Times/week	109	2.720 (1.918–4.412)	2.950 ± 1.888		
≥ 3 Times/week	46	3.492 (2.369–6.645)	3.681 ± 2.069		
Irregular	110	2.947 (1.905–4.073)	2.883 ± 1.891		

a,b,c,d: The differences in hair cortisol concentration between groups with the same symbols are not statiscally significant

As shown in Table 5, HCC levels in two-shift (GMC ± GSD = 3.487 ± 1.930 ng/g) and four-shift (GMC ± GSD = 3.143 ± 1.720 ng/g) groups were significantly higher than those in the fixed day shift (GMC ± GSD = 2.625 ± 2.012 ng/g) and three-shift (GMC ± GSD = 2.994 ± 1.813 ng/g) groups. Four blood models were created to perform a logistic regression analysis on the association between the concentration of hair cortisol and the occurrence of dyslipidaemia at baseline. The results showed that a higher concentration of hair cortisol would lead to an increase in the risk of dyslipidaemia, and the RR (95%CI) was 1.244 (1.102–1.405), *P* < 0.001. For each additional unit of HCC, the risk of dyslipidaemia increased by 27.1, 23.2, 24.0 and 24.4% in Models 1, 2, 3, and 4, respectively (Table 6).

**Table 5:** Differences in hair cortisol concentrations of workers under different shift patterns in Karamay, Xinjiang in 2013

Shift Work	N	HCC M (Q_1_-Q_3_) (ng/g)	HCC GM ± GSD (ng/g)	F	*P*
**Fixed day shift**	127	2.500 (1.612–4.107)	2.625 ± 2.012^a^	2.822	0.041
**Two shifts**	62	3.333 (2.135–5.378)	3.487 ± 1.930^b^		
**Three shifts**	51	2.735 (2.143–4.272)	2.994 ± 1.813^a^		
**Four shifts**	91	3.051 (2.099–4.556)	3.143 ± 1.720^b^		

^a, b^: The differences between groups with the same symbols are not statistically significant

**Table 6:** Relationship between HCC and dyslipidaemia in Karamay City, Xinjiang in 2013

	Dyslipidemia	RR (95%CI)	***p***
**HCC**	**Model 1**	1.271 (1.148–1.407)	< 0.001
	**Model 2**	1.232 (1.102–1.377)	< 0.001
	**Model 3**	1.240 (1.100–1.398)	< 0.001
	**Model 4**	1.244 (1.102–1.405)	< 0.001

Model 1: Represent the association between HCC and dyslipidaemia without the adjustment of confounding factors

Model 2: Adjusted for sex, age, ethnicity, marital status, education level, type of work, length of service, and average monthly income

Model 3: Adjusted for smoking, drinking, physical exercise, and BMI based on Model 2

Model 4: Adjusted for family history of hypertension, coronary heart disease, stroke, and diabetes based on Model 3

**Fig. 1:**
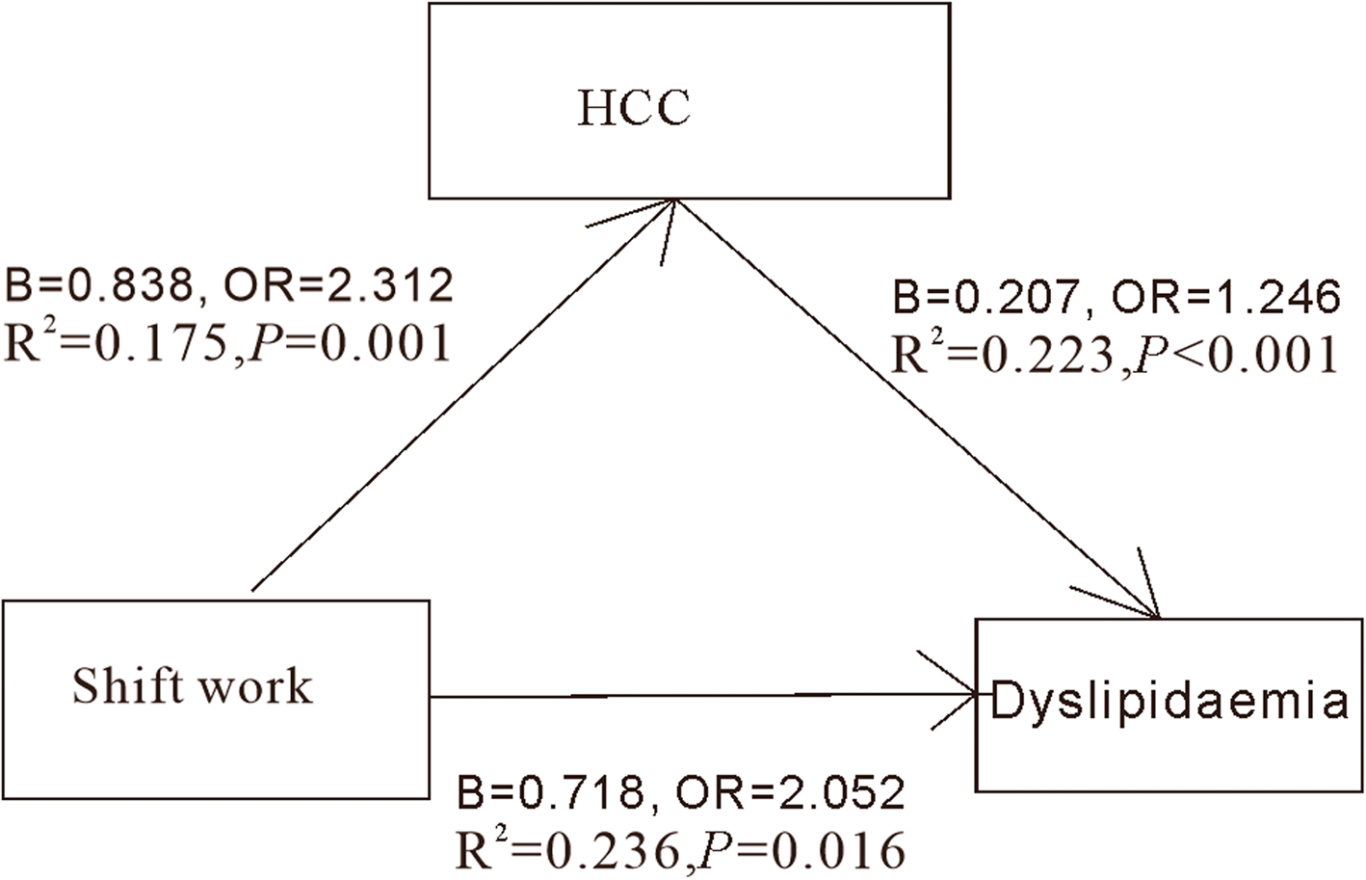
The mediation effect of HCC between shift work and dyslipidaemia. The direction of the arrow in the picture represents the direction of influence. Shift work points to dyslipidaemia represents shift work had an effect on dyslipidaemia. Shift work points to HCC represents shift work had an effect on HCC. HCC points to dyslipidaemia represents HCC had an effect on dyslipidaemia. B: regression coefficient, OR: odds ratio, R^2^: coefficient of determination, P: probability, HCC: hair cortisol concentration, <: less than, =: equal

We used the method of Carlson, Holm, and Brin to evaluate the mediating role of HCC in shift work and dyslipidaemia. The analysis of the mediating effect showed that the regression coefficients of the association between shift work and dyslipidaemia (B = 0.858, 95% CI: 0.271–1.445, OR = 2.359, *P* < 0.05), shift work and HCC (B = 0.838, OR = 2.312, *P* < 0.05), and HCC and dyslipidaemia (B = 0.207, OR = 1.246, *P* < 0.001) were all significant. When HCC was added as a mediator, the regression coefficient remained significant (B = 0.718; 95% CI, 0.133–1.304; OR = 2.052), and the mediation effect of HCC was 0.139 (95% CI = 0.002–0.276, OR = 1.149). We found that HCC played a partial mediating role between shift work and dyslipidaemia; the mediating role was significant, and the mediating effect accounted for 16.24% of the group differences (Table 7 and Fig. 1).

**Table 7:** The mediation effect of HCC between shift work and dyslipidemia in Karamay City, Xinjiang from 2013 to 2019

	Dyslipidemia	***Β*** (95%CI)	SE (β)	z	OR (95%CI)	***p***	(%)
**Shift work**	**Total effect**	0.858 (0.271–1.445)	0.299	2.87	2.359 (1.311–4.242)	0.004	16.24
	**Direct effect**	0.718 (0.133–1.304)	0.298	2.41	2.052 (1.142–3.685)	0.016	
	**Indirect effect**	0.139 (0.002–0.276)	0.070	1.96	1.149 (1.002–1.321)	0.049

## Discussion

We investigated the relationship between shift work, HCC, and dyslipidaemia and explored the effect of HCC as a mediator. We found that the incidence of dyslipidaemia and HCC was higher in shift workers than those in workers with fixed day-shift schedules. We also found that high HCC can lead to a high incidence of dyslipidaemia. HCC played a partially mediating role in the association between shifts and dyslipidemia, and the mediating effect accounted for 16.24% of the relationship.

Relative to day shifts, shift work increases the incidence of dyslipidaemia and is a risk factor for dyslipidaemia. Joo et al. [44] found that night workers had a higher probability of dyslipidaemia than day workers and that there was an association between night work and dyslipidaemia in men but not in women. A subgroup analysis of white-collar workers found that those who worked at night had a higher risk of dyslipidaemia than their daytime working counterparts. We found no differences in the incidence of dyslipidaemia between the two-, three-, and four-shift groups.

The HCC content of workers with two-, three-, and four-shift work patterns was higher than that of regular day shift workers. A study of junior physicians [45] found that waking cortisol levels were significantly higher in shift workers than in non-shift workers. Janssens H [46] et al. found that shift workers had a significantly lower mean HCC than day workers, which was inconsistent with our study findings. A healthy worker effect explained the differences, as their sample of shift workers included workers with a high tolerance for shift work. High HCC leads to a high incidence of dyslipidaemia. However, differences in HCC in the three-shift versus fixed-day shift group and in the four-shift versus two-shift groups were not significant. This is because chronic circadian rhythm disorders reduce plasma cortisol levels [47], and the frequency of four-shift shifts has a large impact on circadian rhythms.

We found that high HCC resulted in a high incidence of dyslipidaemia; after controlling for confounding factors, high HCC was a risk factor for dyslipidaemia. The results differed from the findings of Bancos et al. [31] because of their small sample size or differences in assay methods. Mazgelytė et al. [40] found that an increased prevalence of traditional cardiovascular risk factors is associated with increased HCC. A cross-sectional survey [48] of elderly patients with depression found that high 24-hour urinary cortisol levels were associated with the presence of metabolic syndrome, which included dyslipidaemia. Veen et al. [49] found that in patients with depressive and/or anxiety disorders, elevated basal cortisol concentrations and low circadian cortisol variability were independently associated with higher scores on the lipid index (Lipid index = mean score of the individual z scores for triglycerides, LDL cholesterol, and inverse HDL cholesterol, adjusted for sex and use of oral contraceptives [50]).

We further explored this relationship and found that HCC actually took part in mediating the association between shift work and dyslipidaemia. The mediating effect of HCC accounted for 16.24% of this relationship. At present, there is no relevant research showing a mediating effect between work shift and dyslipidaemia in HCC. We speculate that one possible mechanism is circadian rhythm. Night shift work is associated with disrupted melatonin production [51]. Melatonin can reduce salivary cortisol levels in haemodialysis patients at night [52]. Cortisol is a key player in the circadian system [53] and a critical secondary messenger between the central clock and all peripheral clocks [54]. Clock and Nocturnin, proteins involved in circadian regulation, play important roles in the regulation of dietary lipid absorption [55].

This study had several strengths. First, this is the first study to investigate the mediating effect of HCC on dyslipidaemia in shift workers. Second, we used HCC to reflect long-term cortisol exposure, which was significant in the aetiology of chronic diseases related to HPA axis activation. Third, cohort studies can directly reveal causality. Fourth, dividing workers into different groups according to their shift patterns allowed us to describe the risk of dyslipidaemia associated with different shift patterns. However, this study had some limitations. First, the study participants were oil workers, and our conclusions may not apply to the general population or other workplaces. Second, confounding factors that might have had an important impact on the results were not considered, such as emotion, psychology, consciousness, or depression which were not considered in our study and other issues. Third, with regard to HCC, we only considered the baseline and did not consider the change in HCC over time, which may have affected the aetiology discussion relationship.

## Conclusions

This study found that shift work led to a higher incidence of dyslipidaemia and higher HCC levels than fixed day-shift work. The differences in the incidence of dyslipidaemia among workers with two-, three-, and four-shift schedules were not significant. High HCC levels can cause dyslipidaemia, and HCC had a mediating effect on dyslipidaemia in shift workers.

## Data Availability

The datasets used and/or analysed during the current study are available from the corresponding author on reasonable request.

## Abbreviation

HCC: Hair cortisol concentration
